# Masculinizing hormone therapy effect on breast tissue: Changes in estrogen and androgen receptors in transgender female-to-male mastectomies

**DOI:** 10.1016/j.breast.2023.103596

**Published:** 2023-11-03

**Authors:** Manita Chaum, Sara Grossi, Jiaxi Chen, Vivian Hu, Edward Ray, Armando Giuliano, Shikha Bose

**Affiliations:** a Department of Pathology, Cedars-Sinai Medical Center, Los Angeles, CA, United States; b Division of Plastic & Reconstructive Surgery, Department of Surgery, Cedars-Sinai Medical Center, Los Angeles, CA, United States; c Saul and Joyce Brandman Breast Center, Samuel Oschin Comprehensive Cancer Institute, Cedars-Sinai Medical Center, Los Angeles, CA, United States; d Department of Surgery, University of California San Diego, San Diego, CA, United States

**Keywords:** Gender affirming mastectomies, Exogenous androgen, Breast cancer, Transgender health

## Abstract

**Purpose:**

Almost two percent of individuals in the United States identify as gender non-conforming. In the female-to-male (FTM) transgender population, masculinizing hormone therapy with testosterone is commonly prescribed in gender transition. To date, the effects of exogenous androgens on breast tissue and its roles in altering breast cancer risk are poorly understood. This study examines the histopathologic findings in gender affirming mastectomy (GAM) in transgender FTM patients and the effects of exogenous androgens on estrogen receptors (ER) and androgen receptors (AR).

**Methods:**

A retrospective review of pathology specimens obtained between 2017 and 2020 was performed comparing androgen exposed breast tissue with breast tissue without androgen exposure. Breast specimens were obtained from patients who underwent FTM GAM with recorded exogenous androgen exposure. Control breast specimens were obtained from reduction mammoplasty (RM) procedures in cisgender women which were aged matched to the GAM cohort, as well as postmenopausal women with benign/prophylactic mastectomy procedures; all controls were without androgen exposure. The histopathologic findings were assessed. Immunohistochemistry for AR and ER was performed and the score interpreted by digital image analysis.

**Results:**

Androgen-exposed breast tissue revealed dense fibrotic stroma, lobular atrophy, thickened lobular basement membranes, and gynecomastoid changes. Longer duration of androgen exposure resulted in a more pronounced effect. The incidence of atypia or cancer was lower in GAM than RM cohort. ER and AR expression was highest in transgender male breast tissue with intermediate duration of exogenous androgen exposure.

**Conclusion:**

Increased androgen exposure is associated with lobular atrophy and gynecomastoid changes in breast parenchyma. Overall, ER and AR are expressed strongly in lobular epithelium in patients with prolonged androgen exposure. Exogenous testosterone does not appear to increase risk for breast cancer. Additional studies are needed to investigate the mechanism responsible for these changes at a cellular level and its role in cancer development.

## Highlights

• Exogenous androgen is associated with gynecomastoid changes in breast parenchyma.
• Hormone receptors are expressed strongly with prolonged androgen exposure.
• Exogenous androgen does not appear to increase risk for breast cancer in our cohort.
• Standardized sampling protocol can decrease cost and burden on pathologists.

## 1 Introduction

Gender non-conformity is defined as incongruence between a patient's sex assigned at birth and their gender identity [1]. In 2022, an estimated 1.6 % of the U.S. population identified as transgender [2]. While many people who are gender non-conforming identify as transgender male or female, others may also identify non-binary, gender queer, gender fluid, or any number of other gender identities. Gender dysphoria is the psychological distress associated with this incongruence [1]. Not all people who are gender non-conforming experience gender dysphoria, but many do, causing these individuals varying degrees of distress and often severely affecting quality of life. Patients who experience gender dysphoria are shown to be at increased risk of mood disorders such as anxiety and depression, substance abuse, discrimination, housing insecurity, and domestic abuse [3,4].

Gender dysphoria can be alleviated or reduced through multidisciplinary care, including psychosocial support, hormone therapy, and sometimes gender-affirming surgery [1]. Transgender men are assigned female sex at birth, but identify themselves as male. For transmasculine individuals, gender-affirming therapy often involves administration of exogenous testosterone and a wide variety of gender-affirming procedures such as chest masculinization, metoidioplasty, and/or phalloplasty. The World Professional Association for Transgender Health (WPATH) recommends transgender men socially transition and receive androgen therapy prior to gender-affirming mastectomy [5].

As an increasing number of patients use androgens as part of their gender dysphoria management, the effects of testosterone on breast tissue without exogenous testosterone exposure require further exploration especially in the aspect of altering breast cancer risks. It has previously been shown that breast tissue from transgender men treated with testosterone exhibits lobular atrophy, a reduction in glandular tissue, proliferation of fibrous connective tissue and diminished incidence of atypical hyperplasia or carcinoma in situ compared to controls [1]. Only one study utilized immunohistochemical staining of estrogen, androgen and progesterone receptors in twenty FTM patients without further elaborations on their findings [6]. Otherwise, these tissues have not previously been comprehensively examined for changes in hormone receptors expression.

Hormone receptor expression, including estrogen and progesterone, is often quantified as part of breast profiling in breast cancers (BC). Recently, associations between androgen receptor expression and BC prognosis as well as risk of developing BC have been identified in cisgender women [7,8]. Many studies have also reported increased risk of breast cancer in postmenopausal women due to peripheral conversion of higher baseline endogenous testosterone level to estrogen [5,9,10]. Despite a greater understanding of the implications of hormone receptors in BC, expressivity of steroid hormone receptors has not been thoroughly studied in patients receiving exogenous sex hormones such as testosterone. This study reports androgen and estrogen receptor expression levels in breast tissues of GAM individuals with varying duration of testosterone exposure through histologic sections.

## 2 Materials and methods

The current study protocol, including access to medical records, was approved by the Cedars-Sinai Institutional Review Board (Los Angeles, California). A retrospective review of female-to-male gender affirming mastectomies was performed from the electronic medical record between October of 2017 and May of 2020. All patients provided consent to having their breast specimen examined for our research study. Sixty-four cases of GAM were identified. All patients had testosterone therapy (TT) for at least one year prior to GAM. A control group of sixty-four age-matched cisgender women who underwent reduction mammoplasty (RM) and ten post-menopausal women with breast cancer and contralateral prophylactic mastectomy were also identified. Patient demographics and duration of TT were recorded (Table 1, Table 2). A retrospective review of pathology slides was performed including number of sections submitted, and histopathologic findings (ie. atrophic features and intralobular collagenized stroma and presence of atypia (ie. flat epithelial atypid (FEA), atypical ductal hyperplasia (ADH), atypical lobular hyperplasia (ALH), carcinoma in situ, and invasive carcinoma) were recorded.

**Table 1:** Baseline patient demographics.

Patient Factors	Number of Patients (%)	Gender Affirming Mastectomy	Reduction Mammoplasty	Post Menopausal
Patients included in Study	142 (100 %)	66	66	10
Age (years)				
18 to 40	120 (84.5 %)	62	58	0
41 to 60	17 (12.0 %)	4	8	5
61 to 80	5 (3.5 %)	0	0	5
Mean age	29.1	26.4	26.7	61.6
Race & Ethnicity				
Caucasian, non-hispanic	67 (47.2 %)	31	28	8
Caucasian, hispanic	18 (12.7 %)	9	8	1
Asian	6 (4.2 %)	5	1	0
African American	11 (7.7 %)	4	6	1
Other	40 (28.2 %)	17	23	0
Body Mass Index (BMI) (kg/m^2^)				
<20 to 25	67 (47.2 %)	30	32	5
26 to 35	58 (40.9 %)	29	24	5
>35	17 (12.0 %)	7	10	0
Mean BMI	27.3	28.2	25.9	27.5

**Table 2:** GAM specimens with varying duration of exogenous testosterone therapy (TT) did not show increased incidence of atypia and/or malignancy when compared with age-matched reduction mammoplasty and postmenopausal groups.

Cohorts	Number of patients	Mean age (years)	Average breast sections per case	Number of cases with atypia (%)	Number of cases with malignancy (%)
**Gender affirming mastectomy**	66	26.4	30	1 (1.5 %)	0
1–23 months of TT	43				
24–47 months of TT	10				
48 months of TT	3				
No usage of TT	10				
**Reduction mammoplasty**	66	26.7	10	3 (4.5 %)	0
**Post-menopausal**	10	61.6	Not evaluated	Not evaluated	0

Immunohistochemistry for estrogen receptor (ER), progesterone receptor (PR), and androgen receptor (AR) were performed on a representative section from sixty-four cases: forty-two GAMs with varying testosterone exposure (group 1 included patients with 12–23 months of TT, group 2, patients with 24–47 months of TT and group 3, patients with >48 months of TT), twelve cisgender RM without TT, and ten post-menopausal cisgender female breast tissue without TT. The overall positivity rate was defined as the percentage (0–100 %) of lobular epithelium cells expressing nuclear immunoreactivity of ER, PR and AR within five lobules. The ER, PR and AR staining intensity were categorized into four categories from zero to three (0–3); with zero being absence of nuclear staining and three being the brightest nuclear staining. Both positivity rate and intensity were assessed by digital image analysis with the final scores being an average between five lobules of each specimen. Ductal epithelium was not assessed. Adipocytes and stromal cells were reviewed under the light microscope. The immunohistochemical stains for adipocytes and stromal cells were scored visually as either exhibiting nuclear positivity or negativity by two pathologists.

### 2.1 Immunohistochemistry

The paraffin blocks (n = 64) were cut in 4 μm sections and incubated with ER (Ventana clone SP1), PR (Ventana clone 1E2) and AR (Cell marque clone SP107). Staining was done on the Roche Ventana Benchmark Ultra (Tucson, AZ) automated slide stainer using onboard heat-induced epitope retrieval method in high pH buffer. All slides were subsequently counterstained with Mayer's hematoxylin. The staining for the ER, PR and AR were visualized using the Ventana ultraview DAB Detection System.

### 2.2 Digital image analysis

After proper staining, the glass slides were digitally scanned at 20× and stored into Aperio eSlide Manager software for Windows 7. The clinical laboratory scientists retrieved the digital slides and analyzed five lobules which were previously circled for Aperio Image Scope by using the internally validated ER and PR algorithms to score ER and PR, respectively. Since there is no in-house validated AR algorithm, we utilized ER algorithm to analyze AR immunohistochemistry. The parameters for nuclear inputs include positive intensity threshold, cytoplasmic intensity, remove light objects, clear area intensity, averaging radius, curvature threshold, min/max nuclear size, minimum roundness, minimum compactness, and minimum elongation.

## 3 Results

### 3.1 Histopathologic changes in GAM with prolonged testosterone therapy

In comparison to age-matched RM, pathologic review of GAM tissue revealed predominantly dense fibrotic stroma, lobular atrophy, thickened lobular basement membranes, and gynecomastoid changes characterized by increased periductal fibrosis and decreased lobular epithelial proliferation. Findings were more marked in group three (patients with greater than 48 months of TT).

ADH was seen in one of the sixty-four cases of GAM (1.6 %). The ADH was present unilaterally and was associated with bilateral atypical lobular hyperplasia (ALH). In contrast two cases demonstrated unilateral ADH and one case FEA in the RM cohort (4.7 %). Incidence of atypia was much lower in the GAM group, despite a greater number of sections (2.5 times more) being examined.

#### 3.1.1 Results of hormone receptor analysis

Within the GAM groups 1, 2 and 3, estrogen receptor (ER) and androgen receptor (AR) positivity rate and intensity were highest in group 2 (Fig. 1, Fig. 2). A spectrum of positivity rate varying from 0 to 3 and intensity (varying from 0 to 3) was observed with ER and AR immunohistochemistry, respectively (Fig. 3, Fig. 4). Overall testosterone cohort exhibit higher ER and AR positivity rate and intensity when compared to RM and post-menopausal control groups. In addition, stromal cells in groups 2 and 3 were noted to stain positive for AR (Fig. 5). This is also demonstrable by AR immunohistochemistry (Fig. 6). Progesterone receptor (PR) positivity rate is also highest in group 2. However, PR intensity decreases with prolonged testosterone exposure (Supplement Fig. S1).

**Fig. 1:**
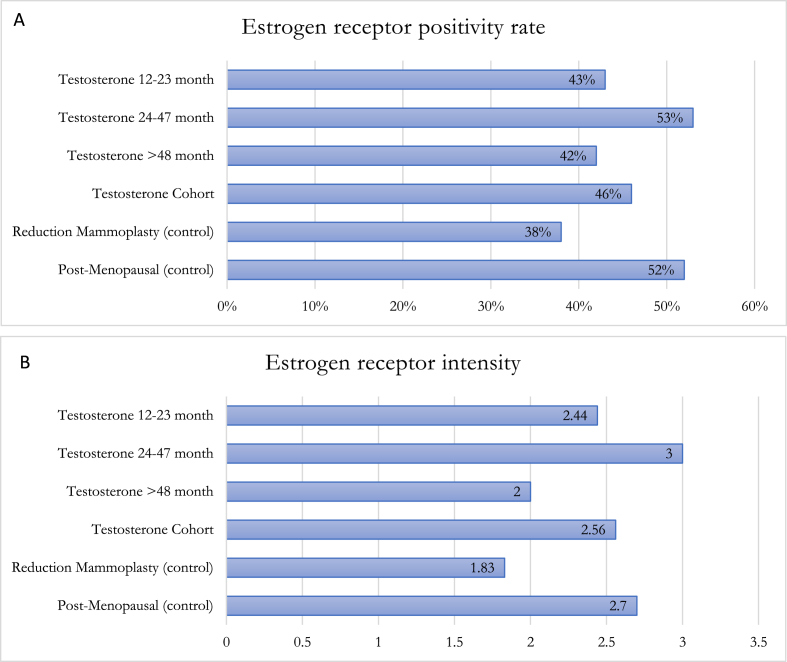
Immunohistochemistry estrogen receptor (A) positivity rate, (B) intensity.

**Fig. 2:**
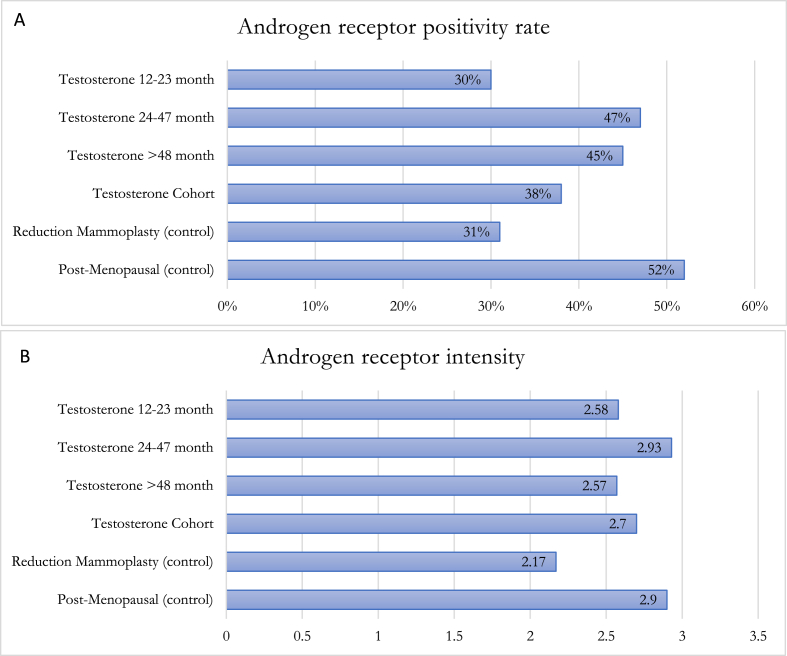
Immunohistochemistry androgen receptor (A) positivity rate, (B) intensity.

**Fig. 3:**
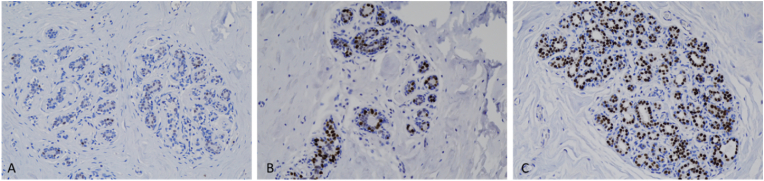
(A) Representative lobule of Group 1 stained by Estrogen Receptor immunohistochemistry with positivity rate of 21 % and intensity of 1. (B) Representative lobule of Group 2 stained by Estrogen Receptor immunohistochemistry with positivity rate of 42 % and intensity of 2. (C) Representative lobule of Group 3 stained by Estrogen Receptor immunohistochemistry with positivity rate of 51 % and intensity of 3. (ER, 20×).

**Fig. 4:**
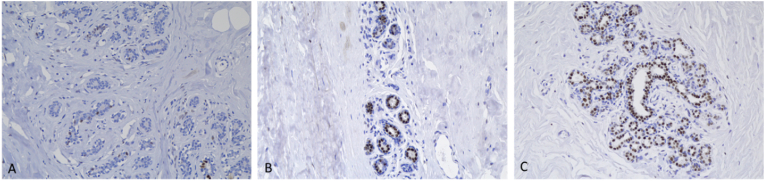
(A) Representative lobule of Group 1 stained by Androgen Receptor immunohistochemistry with positivity rate of 14 % and intensity of 1. (B) Representative lobule of Group 2 stained by Androgen Receptor immunohistochemistry with positivity rate of 52 % and intensity of 2. (C) Representative lobule of Group 3 stained by Androgen Receptor immunohistochemistry with positivity rate of 42 % and intensity of 3. (AR, 20×).

**Fig. 5:**
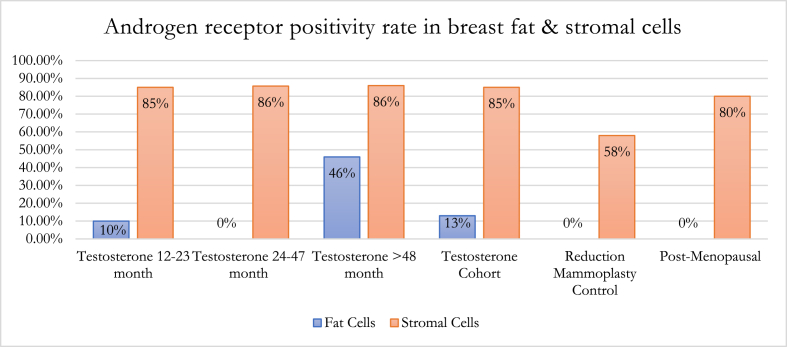
Androgen receptor percent positive in breast fat and stromal cells.

**Fig. 6:**
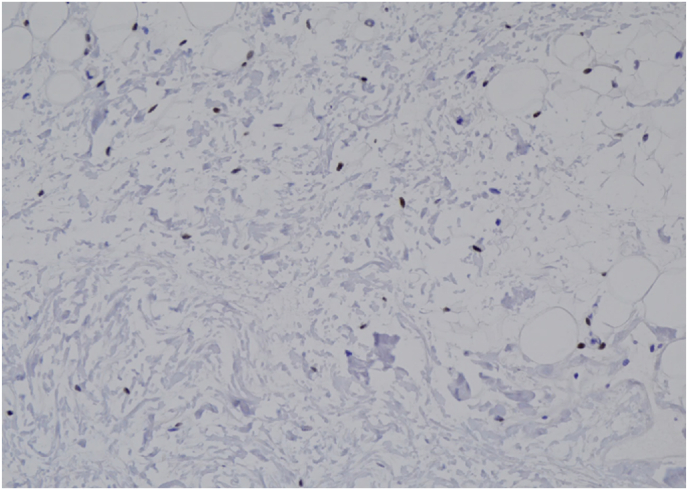
Representative stromal cells of group 3 showing Androgen Receptor nuclear immunoreactivity. (AR, 20×).

## 4 Discussion

An estimated 1.6 % of the US population is identified as transgender. In recent years there is an increase in the number of GAMs. Currently, there are no standard guidelines for examination and reporting of these specimens. Our review of sixty-four GAM breast tissue revealed lower incidence of atypia in comparison to age-matched RM control cohort despite submitting 2.5 times more sections. One case (1.6 %) of bilateral atypical lobular hyperplasia from GAM specimen was noted in comparison to three cases (4.7 %) of atypia in RM control cohort. Previous studies have reported incidence of atypia withing GAM specimen to be between 1.5 % and 3 % [7,[10], [11], [12]]. Neither carcinoma in situ or invasive carcinomas were found in either group. Although the control RM group was age-matched, we did not correlate with other confounding factors such as BMI and family history. The lower incidence of atypia begs the question if pathology laboratories are submitting GAM specimen sections in a cost-effective manner. As mentioned earlier, the pathologists at our institution are reviewing an average of 2.5 times more sections than cisgender RM control cohort. Another study also reported increased anatomic pathologist burden on GAM specimens where an average of 2.8 times more sections are being examined than the cisgender RM sections, and a reported 2.5 times lower significant findings in GAM breast tissue [7].

The incidence of breast cancer in transgender population receiving hormone treatment is not well known with contradicting hypotheses within transgender population and the physiological role of androgen in cisgender females. In both transgender women and men, the reported risk of breast cancer is lower than in cisgender women, suggesting that hormone treatment alters the risk of breast cancer in transgender people compared with baseline risk associated with sex assigned at birth. This was additionally confirmed by Kensler et al. and de Blok et al. [9,13].

On the contrary through extrapolating data from postmenopausal women, it is observed that high baseline serum testosterone is a strong prognostic factor for local relapse, contralateral BC, and distant metastases. High levels of other androgens (free testosterone, DHEAS, and androstenedione) and SHBG (steroid hormone binding globulin) are correlated with an increased post-menopausal BC risk [[14], [15], [16], [17]]. Several studies have attempted to evaluate the role of androgens in breast cancer etiology. One prospective cohort was included in the Nurses' Health Study, observed from 1978 to 2002 to assess the risk of breast cancer associated with different types of postmenopausal hormone (PMH) formulations containing testosterone. The authors concluded that the risk of breast cancer associated with the use of estrogen and testosterone therapy was significantly greater compared with estrogen-only therapy [18]. Other epidemiologic studies suggest that endogenous androgen levels are positively associated with breast cancer risk. A combined reanalysis of data from nine prospective studies investigating the association between endogenous hormone levels and risk of breast cancer reported that testosterone, androstenedione, dehydroepiandrosterone, and dehydroepiandrosterone sulfate were all associated with increased risk of breast cancer [18,19]. The physiologic basis for this increase in the risk of breast cancer from exogenous androgens may be related to the conversion of androgens to estrogens or more directly with effects mediated through the androgen receptor [18,20,21]. Specifically, testosterone may serve as a substrate for local estradiol formation in the breast because breast tissue has aromatase activity [10,21]. While further studies are warranted, our result does not support the hypothesis that exogenous androgen correlates with an increased BC risk. Of note, we also cannot assume that the roles of endogenous androgen is similar to exogenous androgen in altering BC risk.

Pathologic changes in breast tissue were related to the duration of testosterone therapy. Lobular atrophy and stromal fibrosis were the prominent findings in our cases. Similar studies also noted that the degree of lobular atrophy correlated with the duration of TT [[14], [15], [16], [17]]. One study sought to analyze the androgen and estrogen receptor “crosstalk” in cancer [22]. Because the androgen rector (AR) is a transcription factor that binds specific androgen response elements on DNA, it is located in the cytoplasm in its inactive state and is bound to heat shock proteins (HSPs). Upon androgen stimulation, the AR is released from these HSPs where it translocates into the nucleus and subsequently regulates androgen-responsive genes [23]. Similarly, exogenous androgen could undergo aromatase conversion to estrogen causing increased ER activation within breast tissue. This may account for the observed increased levels of both ER and AR positivity rate and intensity in pathologic samples corresponding with duration of TT exposure. One study reported transgender male breast tissue to exhibit consistent positivity and strong intensity for ER, PR and AR by immunohistochemistry [6]. This result is congruent with our study where the comparison between the 12–23 months and 24–47 months of TT cohort shows an increased number of lobular epithelium cells exhibiting ER positivity (ie. ER positivity rate) from 43 % to 53 %; whereas AR positivity rate increase from 33 % to 47 %. Within the same cohort, ER and AR express brighter nuclear staining with an increasing score from 2.44 to 3 and 2.58 to 2.93, respectively. Interestingly, both ER and AR positivity rate and intensity decrease at >48 months TT. This could indicate a transient increase in hormone receptors levels at 24–47 months and subsequent readjustment after some duration of TT exposure. Interestingly, within our transgender male breast tissue previously exposed to TT, the ER and AR positivity rate and intensity are lower than the post-menopausal control group, but higher than the age-matched cisgender female reduction mammoplasty control group. When compared to both control groups, whether the intermediate findings of both hormone receptors in transgender men can be utilized in breast cancer risk stratification warrant additional studies [[14], [15], [16], [17]].

One study sought to analyze the androgen and estrogen receptor “crosstalk” in cancer [22]. Because the androgen receptor (AR) is a transcription factor that binds specific androgen response elements on DNA, it is located in the cytoplasm in its inactive state and is bound to heat shock proteins (HSPs). Upon androgen stimulation, the AR is released from these HSPs where it translocates into the nucleus and subsequently regulates androgen-responsive genes [23]. Similarly, exogenous androgen could undergo aromatase conversion to estrogen causing increased ER activation within breast tissue. This may account for the observed increased levels of both ER and AR positivity rate and intensity in pathologic samples corresponding with duration of TT exposure. One study reported transgender male breast tissue to exhibit consistent positivity and strong intensity for ER, PR and AR by immunohistochemistry [6]. This result is congruent with our study where the comparison between the 12–23 months and 24–47 months of TT cohort shows an increased number of lobular epithelium cells exhibiting ER positivity (ie. ER positivity rate) from 43 % to 53 %; whereas AR positivity rate increase from 33 % to 47 %. Within the same cohort, ER and AR express brighter nuclear staining with an increasing score from 2.44 to 3 and 2.58 to 2.93, respectively. Interestingly, both ER and AR positivity rate and intensity decrease at >48 months TT. This could indicate a transient increase in hormone receptors levels at 24–47 months and subsequent readjustment after some duration of TT exposure. Interestingly, within our transgender male breast tissue previously exposed to TT, the ER and AR positivity rate and intensity are lower than the post-menopausal control group, but higher than the age-matched cisgender female reduction mammoplasty control group. When compared to both control groups, whether the intermediate findings of both hormone receptors in transgender men can be utilized in breast cancer risk stratification warrant additional studies.

Further investigation is needed to elucidate the clinical significance of the histopathologic findings of TT and increased AR expression on breast tissue at the cellular level [24,25]. For example, although AR expression is often associated with a favorable prognosis in ER-positive BC, high levels of AR can also contribute to therapy resistance [26, 28, 29]. In ER-negative BC, AR is expressed in tumors with apocrine differentiation and lower Nottingham grade and may stimulate cellular proliferation in triple negative breast cancer [27]. More understanding of complex interactions between exogenous testosterone exposure of varying durations, breast hormone receptors expression and their roles in altering breast cancer risks may be a useful component in constructing breast cancer screening guidelines in transgender population.

## 5 Limitations of study

Our analysis was limited by a relatively smaller cohort size especially in >48 months TT GAM group. AR immunohistochemistry assessment by digital image analysis has limitations due to lack of an in-house validated algorithm. Hence, a validated in-house ER algorithm was used to assess AR intensity and positivity rate. Generalizability of this study may be limited given the small sample size of patients who underwent AR testing in the present study. Furthermore, AR stains are not routinely performed on GAM specimens and may be costly.

## 6 Conclusion

Despite more sections being submitted from GAM specimens, the incidence of atypia is lower in GAM in comparison to cisgender age-matched female reduction mammoplasty breast tissue. We recommend an evaluation on a case-by-case basis including careful examination with limited sampling in GAM specimens without gross or radiologic abnormalities, and known breast cancer risk factors to be cost-effective and decrease the burden on anatomic pathologists. Exposure to testosterone causes lobular atrophy and gynecomastoid changes and this histopathologic change intensifies with duration of exposure to androgen. Both ER and AR exhibit highest positivity rate and intensity in lobular epithelium of FTM transgender patients with 24–47 months and subsequently decrease after >48 months of TT exposure. The overall transgender male breast tissue exposed to TT displays lower ER and AR positivity rate and intensity than the post-menopausal control group, but higher than the age-matched cisgender female reduction mammoplasty control group. AR nuclear reactivity in stromal cells also intensifies with duration of TT. Further investigation is warranted to elucidate breast cancer risks in transgender individuals receiving exogenous testosterone of varying durations and its roles in altering ER and AR. Since FTM transgender individuals use exogenous testosterone for extended periods of time and consideration of culturally sensitive routine breast cancer screening protocols for these individuals is paramount, long-term prospective studies and molecular investigations to further understand the breast cancer risk of individuals who receive TT is warranted.

## Financial disclosure

The authors have no financial interests including products, devices, or drugs associated with this manuscript. There are no commercial associations that might pose or create a conflict of interest with information presented in this submitted manuscript such as consultancies, stock ownership, or patent licensing arrangements.

## Author contribution statement

Conceptualization, MC, AG, ER, SB; Data Curation, JC, MC, VH; Formal Analysis, JC, MC, VH, SG; Funding Acquisition: AG, ER, SB; Investigation, JC, VH, MC; Methodology, AG, ER, SB, JC, MC; Project Administration, AG, ER, SB; Resources, AG, ER, SB; Software, JC, VH, MC, SG; Supervision, AG, ER, SB; Validation, JC, MC, SG; Visualization, JC, VH, MC, SG; Writing-Original Draft, SG, JC, VH, MC; Writing-review & editing, SG, JC, MC, AG, ER, SB.

## Funding information

All sources of funds supporting the completion of this manuscript are under the auspices of 10.13039/100013015Cedars-Sinai Medical Center. Funding for the project was provided by grants from Dr. Armando Giuliano and Dr. Randy Sherman.

## Declaration of competing interest

All authors have no relevant conflicts of interest to disclose.

## Figures and Tables

**figs1:**
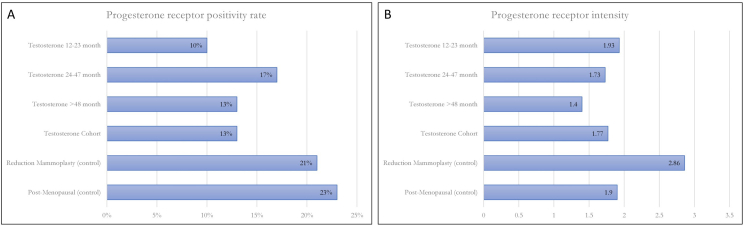


## References

[1] Coleman E., Bockting W., Botzer M. (2012). Standards of care for the health of transsexual, transgender, and gender-nonconforming people, version 7. Int J Transgenderism.

[2] (2022).

[3] Dhejne C., van Vlerken R., Heylens G. (2016). Mental health and gender dysphoria: a review of the literature. Int Rev Psychiatr.

[4] Eckstrand K., Ehrenfeld J. (2016).

[5] Grynberg M., Fanchin R., Dubost G. (2010). Histology of genital tract and breast tissue after long-term testosterone administration in a female-to-male transsexual population. Reprod Biomed Online.

[6] East E.G., Gast K.M., Kuzon W.M. (2017). Clinicopathological findings in female-to-male gender-affirming breast surgery. Histopathology.

[7] Hernandez A., Schwartz C.J., Warfield D. (2020). Pathologic evaluation of breast tissue from transmasculine individuals undergoing gender-affirming chest masculinization. Arch Pathol Lab Med.

[8] Kono M., Fujii T., Lim B. (2017). Androgen receptor function and androgen receptor–targeted therapies in breast cancer. JAMA Oncol.

[9] Baker G.M., Guzman-Arocho Y.D., Bret-Mounet V.C. (2021). Testosterone therapy and breast histopathological features in transgender individuals. Mod Pathol.

[10] East E.G., Gast K.M., Kuzon W.M. (2017). Clinicopathological findings in female-to-male gender-affirming breast surgery. Histopathology.

[11] Torous V.F., Schnitt S.J. (2019). Histopathologic findings in breast surgical specimens from patients undergoing female-to-male gender reassignment surgery. Mod Pathol.

[12] Kensler K.H., Beca F., Baker G.M. (2018). Androgen receptor expression in normal breast tissue and subsequent breast cancer risk. NPJ Breast Cancer.

[13] de Blok C.J.M., Wiepjes C.M., Nota N.M. (2019). Breast cancer risk in transgender people receiving hormone treatment: nationwide cohort study in The Netherlands. BMJ.

[14] Grynberg M., Fanchin R., Dubost G. (2010). Histology of genital tract and breast tissue after long-term testosterone administration in a female-to-male transsexual population. Reprod Biomed Online.

[15] Kensler K.H., Poole E.M., Heng Y.J. (2019). Androgen receptor expression and breast cancer survival: results from the Nurses' health studies. J Natl Cancer Inst.

[16] Kuroda H., Ohnisi K., Sakamoto G. (2008). Clinicopathological study of breast tissue in female-to-male transsexuals. Surg Today.

[17] Baker G.M., Pyle M.E., Tobias A.M. (2019). Establishing a cohort of transgender men and gender nonconforming individuals to understand the molecular impact of testosterone on breast physiology. Transgend Health.

[18] Key T., Appleby P., Barnes I. (2002). Endogenous sex hormones and breast cancer in postmenopausal women: reanalysis of nine prospective studies. J Natl Cancer Inst.

[19] Fledderus A.C., Gout H.A., Ogilvie A.C. (2020). Breast malignancy in female-to-male transsexuals: systematic review, case report, and recommendations for screening. Breast.

[20] Tamimi R.M., Hankinson S.E., Chen W.Y. (2006). Combined estrogen and testosterone use and risk of breast cancer in postmenopausal women. Arch Intern Med.

[21] Esteban J.M., Warsi Z., Haniu M. (1992). Detection of intratumoral aromatase in breast carcinomas. An immunohistochemical study with clinicopathologic correlation. Am J Pathol.

[22] de Blok C.J.M., Wiepjes C.M., Nota N.M. (2019). Breast cancer risk in transgender people receiving hormone treatment: nationwide cohort study in The Netherlands. The BMJ.

[23] Karamouzis M v, Papavassiliou K.A., Adamopoulos C. (2016). Targeting androgen/estrogen receptors crosstalk in cancer. Trends Cancer.

[24] Giovannelli P., di Donato M., Galasso G. (2018). The androgen receptor in breast cancer. Front Endocrinol.

[25] Bulun S.E., Price T.M., Aitken J. (1993). A link between breast cancer and local estrogen biosynthesis suggested by quantification of breast adipose tissue aromatase cytochrome P450 transcripts using competitive polymerase chain reaction after reverse transcription. J Clin Endocrinol Metab.

[26] Labrie F., Luu-The V., Labrie C. (2003). Endocrine and intracrine sources of androgens in women: inhibition of breast cancer and other roles of androgens and their precursor dehydroepiandrosterone. Endocr Rev.

[27] McNamara K.M., Moore N.L., Hickey T.E. (2014). Complexities of androgen receptor signalling in breast cancer. Endocr Relat Cancer.

